# Expression Profiles of mRNA and lncRNA in HCT-8 Cells Infected With *Cryptosporidium parvum* IId Subtype

**DOI:** 10.3389/fmicb.2018.01409

**Published:** 2018-06-27

**Authors:** Ting-Li Liu, Xian-Chen Fan, Yun-Hui Li, Ya-Jie Yuan, Yan-Ling Yin, Xue-Ting Wang, Long-Xian Zhang, Guang-Hui Zhao

**Affiliations:** ^1^College of Veterinary Medicine, Northwest A&F University, Yangling, China; ^2^College of Animal Science and Veterinary Medicine, Henan Agricultural University, Zhengzhou, China

**Keywords:** mRNAs, lncRNAs, microarray, *Cryptosporidium parvum* IId subtype, signaling pathway, regulation

## Abstract

*Cryptosporidium parvum* is one of the most important enteric protozoan pathogens, responsible for severe diarrhea in immunocompromised human and livestock. However, few effective agents were available for controlling this parasite. Accumulating evidences suggest that long non-coding RNA (lncRNA) played key roles in many diseases through regulating the gene expression. Here, the expression profiles of lncRNAs and mRNAs were analyzed in HCT-8 cells infected with *C. parvum* IId subtype using microarray assay. A total of 821 lncRNAs and 1,349 mRNAs were differentially expressed in infected cells at 24 h post infection (pi). Of them, all five types of lncRNAs were identified, including 22 sense, 280 antisense, 312 intergenic, 44 divergent, 33 intronic lncRNAs, and 130 lncRNAs that were not found the relationship with mRNAs’ location. Additionally, real-time polymerase chain reactions of 10 lncRNAs and 10 mRNAs randomly selected were successfully confirmed the microarray results. The co-expression and target prediction analysis indicated that 27 mRNAs were *cis*-regulated by 29 lncRNAs and 109 were *trans*-regulated by 114 lncRNAs. These predicted targets were enriched in several pathways involved in the interaction between host and *C. parvum*, e.g., hedgehog signaling pathway, Wnt signaling pathway, and tight junction, suggesting that these differentially expressed lncRNAs would play important regulating roles during the infection of *C. parvum* IId subtype.

## Introduction

*Cryptosporidium*, one of the most important enteric parasites to cause diarrhea in human and animals (Holland, 1990; Nguyen et al., 2013; Koinari et al., 2014; Delafosse et al., 2015; Deshpande et al., 2015; Nakamura and Meireles, 2015), has been recognized as the leading cause of chronic diarrhea in HIV patients and was the second contributor of moderate-to-severe diarrhea in children during the first 2 years of life (Checkley et al., 2015). After ingested, the sporozoites within *Cryptosporidium* oocysts were released and colonized into epithelial cells of the gastrointestinal tract, damaged the intestinal barriers to affect the host nutrition absorption, impaired immune response, and persistently retarded growth (Guerrant et al., 1999; Gookin et al., 2002; Mondal et al., 2009; Squire and Ryan, 2017).

Currently, 33 valid *Cryptosporidium* species have been confirmed and scattered around the world (Xiao, 2010; Fletcher et al., 2012; Hu et al., 2014; Ryan et al., 2014, 2015; Li et al., 2015; Holubová et al., 2016; Jezkova et al., 2016; Kváč et al., 2016; Feng and Xiao, 2017; Zahedi et al., 2017). Of them, *Cryptosporidium parvum* has been identified as the most common zoonotic species infecting humans and most animals (Wang et al., 2014). Intra-species genetic diversity of *C. parvum* was observed based on the nucleotide sequence of 60 kDa glycoprotein (GP60) gene, and at least 15 subtypes (IIa–IIi, IIk–IIp) were identified (Xiao, 2010; Insulander et al., 2013; Wang et al., 2014). In China, *C. parvum* has been detected in humans and 18 animal species from 19 provinces and one region (Qinling Mountain; Huang et al., 2014; Qi et al., 2015; Zhang X.X. et al., 2015; Cai et al., 2017). Of them, both subtypes IIa and IId were identified in China (Mi et al., 2013; Cui et al., 2014; Zhao et al., 2015). The subtype IId was widely detected in sheep, goats, and calves from China (Feng et al., 2013, 2017). Besides, the whole genome of two subtypes was different in sizes and contents. For example, the total assembly length of *C. parvum* IIaA15G2R1 was longer than IIdA19G1, and the gene gains/losses (e.g., SKSR^a^, MEDLE family of secreted proteins, Insulinase-like proteases) and single nucleotide variants (SNVs) were also found between the genomes of two subtypes (Widmer et al., 2012; Feng et al., 2017).

In the process of *C. parvum* invasion and parasitism, the host–parasite interaction occurred. The expression profiles of both mRNA and non-coding RNA (ncRNA) during the infection of *C. parvum* Iowa isolate (IIa subtype) have been investigated (Abrahamsen et al., 1996; Deng et al., 2004; Zhou et al., 2014). The differentially expressed mRNAs were predicted to be associated with the promoter enrichment of suppressive epigenetic marker, while ncRNAs may modulate epithelial immune responses and epithelial anti-microbial defense against *Cryptosporidium* infection (Abrahamsen et al., 1996; Zhou et al., 2009, 2014; Wang et al., 2017b). However, the gene expression in hosts infected with *C. parvum* subtype IId was not available. Additionally, a new RNA molecule, long non-coding RNA (lncRNA), was discovered and studied recently (Okazaki et al., 2002). Increasing evidences have certificated that lncRNAs could interact with mRNAs through *cis*- and *trans*-regulation to play key roles in tumorigenesis, tumor development (He et al., 2014; Lee et al., 2016), and infections of viruses (e.g., hepatitis C virus, influenza A virus, and severe acute respiratory syndrome coronavirus) and parasites (e.g., *Leishmania*, *Plasmodium falciparum*; Josset et al., 2014; Broadbent et al., 2015; Zhang H. et al., 2015; Pawar et al., 2017). To deeply understand the interaction between *C. parvum* and host, herein, we systematically investigated the expression profiles of mRNA and lncRNA in human cells infected with the *C. parvum* IIdA19G1 subtype.

## Materials and Methods

### *C. parvum* Isolates

The oocysts used in the present study were obtained from a pre-weaned dairy calve with diarrhea in China and molecularly identified as *C. parvum* IIdA19G1 subtype based on the sequence of the gp60 gene locus. This isolate was passaged by pre-weaned dairy calves in the laboratory with the specific pathogen-free condition. *C. parvum* oocysts were purified by using the Sheather’s sugar flotation technique and cesium chloride density gradient centrifugation, and stored in PBS with the penicillin-streptomycin (100 U/ml penicillin and 0.1 mg/ml streptomycin) and amphotericin B solutions (0.25 μg/ml).

### *In Vitro* Infection Model of *C. parvum*

The human adenocarcinoma (HCT-8) cell lines were purchased from JENNIO Biological Technology (Guangzhou, China). 2 × 10^5^ HCT-8 cells were seeded in each well of a fresh 24-well plate and cultured for 24 h (or with 80% confluence) at RMPI 1640 medium and supplemented with 10% fetal bovine serum (FBS) under 5% CO_2_ at 37°C. *C. parvum* oocysts were treated with 2% bleach for 20 min on ice and incubated into HCT-8 cells with the ratio of oocysts: cells = 2–10:1. The infection burden was measured using quantitative real-time polymerase chain reaction (qRT-PCR) targeting the small subunit ribosomal RNA (SSU rRNA) previously described (Zhao et al., 2018).

### Sample Collection, RNA Extraction, and Microarray Analysis

The cell samples were collected from both experimental (*C. parvum* infection, O) and control (without parasites, C) groups at 24 h post infection (pi) of *C. parvum* oocysts. Three biological repeats were included in each group. Total RNA was extracted using TRIzol reagent (500 μl) and the chloroform-isopropyl alcohol method in accordance with the manufacturer’s instructions and stored at −80°C. The concentration and purity of total RNA samples were measured by the Smart Spec Plus spectrophotometer. The complementary DNA (cDNA) was generated using the PrimeScript TM RT reagent Kit with the gDNA Eraser (TaKaRa Shuzo Co., Ltd., Liaoning, China) following the manufacturer’s instructions for reverse transcription of the total RNAs (1 μg). Then, the samples were taken and sent to the company (CapitalBio Technology Corporation, Beijing, China) for microarray analysis. The expression profiles of mRNAs and lncRNAs were detected by using LncRNA+mRNA Human Gene Expression Microarray V4.0 (4 × 180 K). The Agilent Feature Extraction v10.7 was used to analyze and extract data, and these data were then normalized and analyzed by using Agilent GeneSpring GX software (13.1 revisions, 2015^1^).

### GO and KEGG Enrichment Analysis

To investigate the biological functions of mRNAs and lncRNAs, the Gene Ontology (GO) analysis was executed. Three parts were involved in the GO terms, including biological process (BP), cellular component (CC), and molecular function (MF). The enrichment analysis of GO terms with *P* < 0.05 was considered significantly. Besides, Kyoto Encyclopedia of Genes and Genomes (KEGG) was used to test the statistical enrichment of differentially expressed mRNAs and lncRNAs to predict the possible pathways involved. Both GO and KEGG were carried out by KOBAS 2.0 software (2016^2^).

### Validation of Microarray Data by qRT-PCR

In order to validate the microarray results, five up- and five down-regulated genes of both mRNA and lncRNA were, respectively, selected for qRT-PCR validation, with *GAPDH* as an internal control, and the sequences of primers listed in **Supplementary Table S1**. The qRT-PCR was carried out using the SYBR assay in a 10 μl reaction volume, containing 0.2 μl Forward Primer, 0.2 μl Reverse Primer, 1 μl cDNA, 3.6 μl nuclease-free H_2_O, and 5 μl Master Mix (CWBIO, Beijing, China). The reaction program was initiated at 95°C for 10 min, then at 95°C for 10 s, 60°C for 60 s for a total 40 cycles. Three replicates were conducted for each gene, and the data were expressed as 2^−ΔΔCt^ to value the expression of mRNA and lncRNA.

### Statistical Analysis

All statistical analysis in this study was performed using the software GraphPad Prism 7.0 (2016^3^), with *P* < 0.05 considered as statistically significant.

## Results

### Identification of mRNA and LncRNA Differentially Expressed in *C. parvum* Infection Model

**FIGURE 1:**
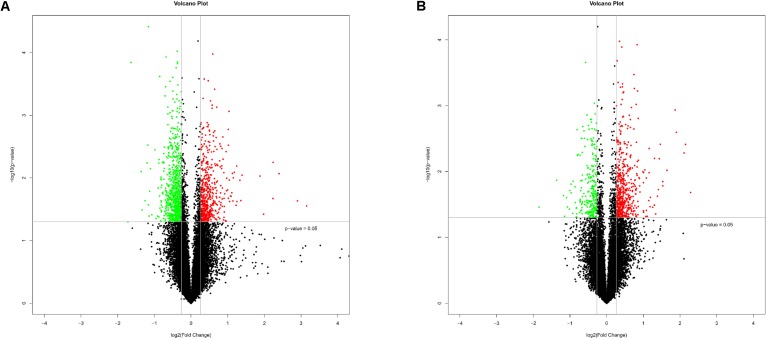
Bioinformatics analysis of differentially expressed mRNAs and lncRNAs in HCT-8 cells infected with *Cryptosporidium parvum* IId subtype. **(A)** The volcano plot shows the distributions of mRNAs. **(B)** The volcano plot shows the distributions of lncRNAs. The significantly up- and down-regulated RNAs are presented as red or green dots, respectively, and the expression of RNAs not significantly differentially expressed is presented as black dots (fold change ≥ 1.2 and *P* < 0.05).

To explore the impact of Chinese prevalent *C. parvum* subtype (IIdA19G1 subtype) on human cells, HCT-8 cells were exposed to *C. parvum* IIdA19G1 subtype for 24 h and collected for LncRNA+mRNA Human Gene Expression Microarray analysis. The result revealed the differential expression profiles of mRNA and lncRNA in HCT-8 cells after *C. parvum* infection. Because of mild influence of *C. parvum* to host cell gene transcription (Deng et al., 2004; Zhou et al., 2009; Ming et al., 2017), the fold change ≥ 1.2 and *P* < 0.05 were used as the standard to map differentially expressed genes. All microarray data obtained from our study were deposited into GEO database with the number GSE111565. The expression patterns of mRNA (**Supplementary Figure S1A**) and lncRNA (**Supplementary Figure S1B**) between experimental and control groups were found to be significantly different by hierarchical clustering plot. A total of 1349 mRNAs (including 535 up- and 814 down-regulated; **Figure 1A** and **Supplementary Table S2**) and 821 lncRNAs (including 557 up- and 264 down-regulated; **Figure 1B** and **Supplementary Table S3**) were found to be differentially expressed by volcano plot and scatter plot filtering (**Supplementary Figure S2**). These lncRNAs were grouped into five types reported previously, including 22 sense, 280 antisense, 312 intergenic, 44 divergent, and 33 intronic lncRNAs. Additionally, 130 lncRNAs that were not found the relationship with mRNAs’ location were also found to be differentially expressed.

### Validation of Differentially Expressed mRNAs and LncRNAs

**FIGURE 2:**
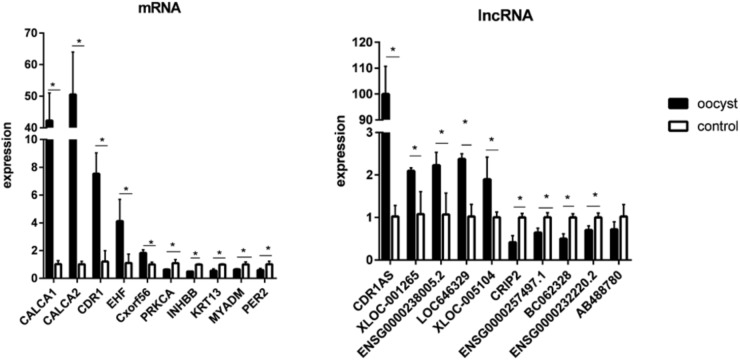
Validation for the expression of 10 significantly differential expressions of mRNAs and 10 lncRNAs by qRT-PCR. Three biological repeats were included in each gene. ^∗^*P* < 0.05.

To validate the microarray data, 10 differentially expressed mRNAs (CALCA1, CALCA2, CDR1, EHF, Cxorf56, PRKCA, INHBB, KRT13, MYADM, and PER2) and 10 lncRNAs (CDR1AS, XLOC_001265, ENSG00000238005.2, LOC636429, XLOC_005104, CRIP2, ENSG00000257497.1, BC062328, ENSG00000232220.2, and AB488780) were randomly selected for qRT-PCR. The consistent results were found between qRT-PCR and microarray data for 10 mRNAs and nine lncRNAs (**Supplementary Figure S3**). Although the difference in the expression of the lncRNA AB488780 was not statistically significant by qRT-PCR, a consistent expression trend of this gene was observed between qRT-PCR and microarray (**Figure 2**).

### GO and KEGG Pathway Analysis of Differentially Expressed mRNAs

**FIGURE 3:**
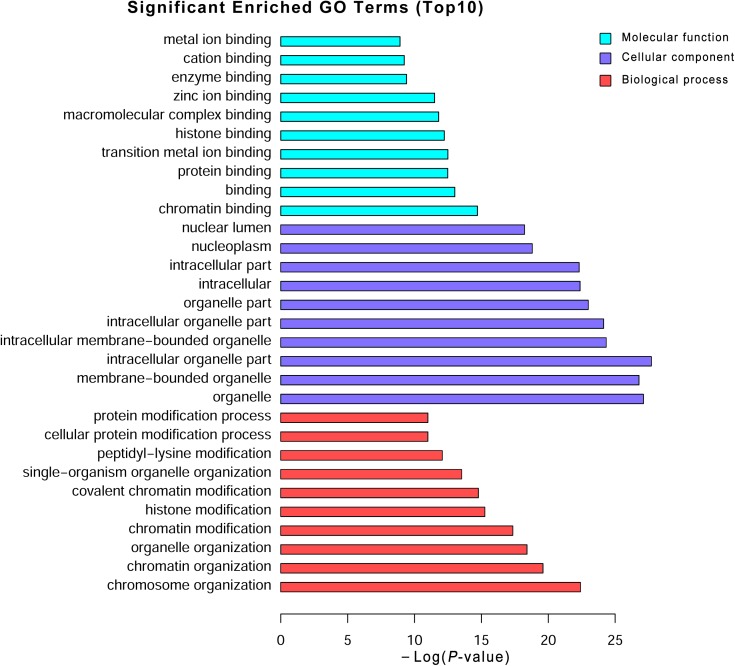
Gene Ontology (GO) analysis of the differentially expressed mRNAs. Go annotation of differentially expressed mRNAs with top 10 enrichment scores covering domains of biological processes, cellular components, and molecular functions. The GO terms with corrected *P*-value less than 0.05 were considered significantly enriched by differentially expressed genes.

**FIGURE 4:**
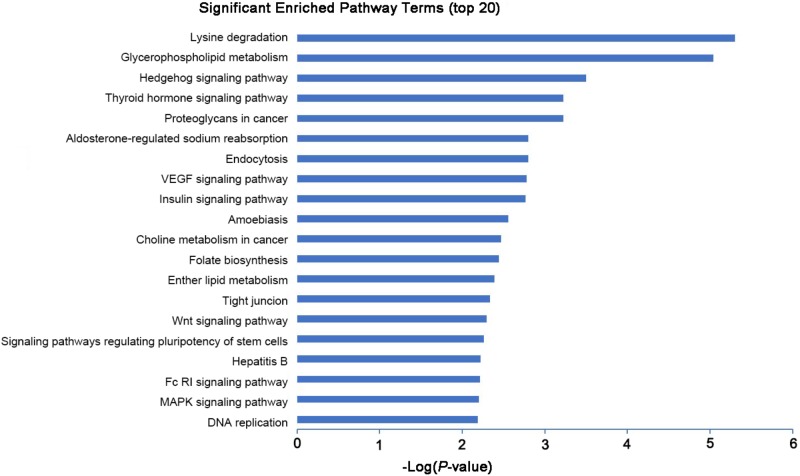
KEGG pathway analysis of the differentially expressed mRNAs with top 20 enrichment scores.

To explore the potential biological functions of the differentially expressed mRNAs, the GO and KEGG pathway enrichments were carried out. The GO analysis indicated that these mRNAs were significantly enriched in chromosome organization, chromatin organization, organelle organization, and chromatin modification in BP; intracellular organelle, organelle, and membrane-bounded organelle in CC; chromatin binding, binding, transition metal ion binding, and protein binding in MF (**Figure 3** and **Supplementary Table S6**). The pathways and molecular interactions associated with significantly differentially expressed mRNAs were then predicted by KEGG pathway enrichment analysis. The top 20 pathways were depicted in **Figure 4**, with a great number of mRNAs enriched into hedgehog signaling pathway, tight junction, and Wnt signaling pathway (**Supplementary Table S7**).

### Co-expression of LncRNA and mRNA

**FIGURE 5:**
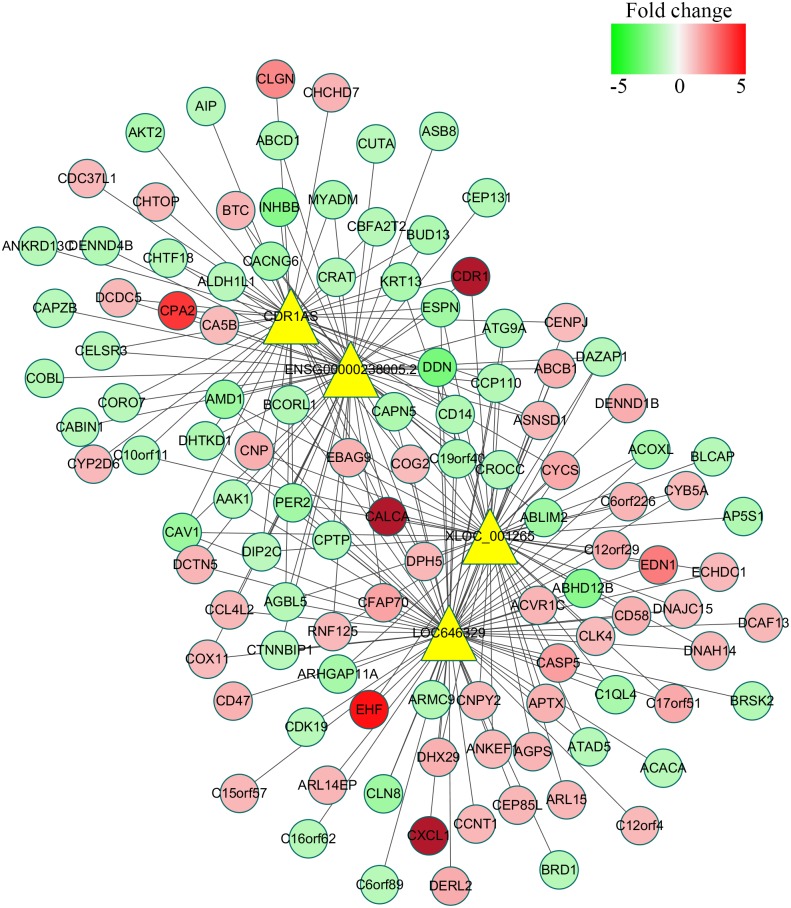
Co-expression network of four significantly differentially expressed lncRNAs with their associate mRNAs. The network was based on the mathematical relevance (Correlation > 0.99, Correlation < –0.99, and *P*-value < 0.05) to search similar expression profiles of lncRNAs and mRNAs using cytoscape software (v3.6.0). The yellow triangle represents lncRNAs while the gradual color of red to green circular represents mRNAs. The black solid line indicates the correction of lncRNAs and mRNAs.

To reveal the correlation between differentially expressed lncRNAs and mRNAs and figure out the possible mechanisms of lncRNAs during *C. parvum* infection, the co-expression network was constructed based on the mathematical relevance (Correlation > 0.99, Correlation < −0.99, and *P*-value < 0.05) to search similar expression profiles of lncRNAs and mRNAs. The co-expression network was constructed by using Cytoscape v3.6.0 (2015^4^). In this network, one mRNA could be correlated with one or more lncRNAs, and one lncRNA could also be associated with one or more mRNAs (**Supplementary Table S4**). For example, the mRNA MYADM was related to the two lncRNAs (CDR1AS and ENSG00000238005.2), and the lncRNA LOC636429 corresponded to more than 300 mRNAs (**Figure 5** and **Supplementary Table S4**).

### Functional Prediction of LncRNAs During *C. parvum* Infection

**FIGURE 6:**
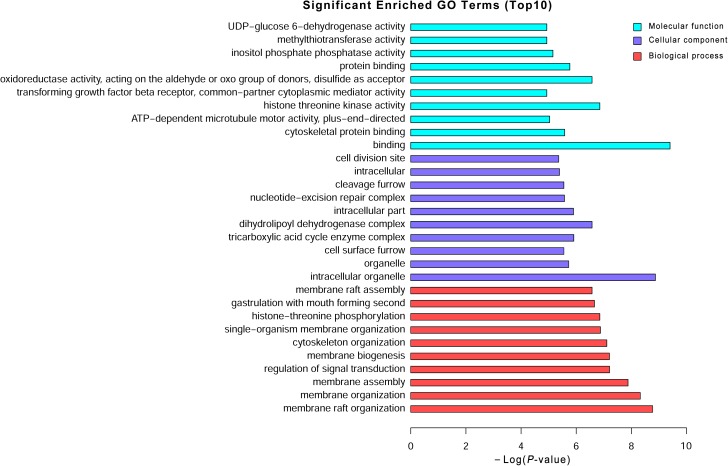
Gene Ontology (GO) analysis of the differentially expressed lncRNAs. Go annotation of differentially expressed lncRNAs with top 10 enrichment scores covering domains of biological processes, cellular components, and molecular functions. The GO terms with corrected *P*-value less than 0.05 were considered significantly enriched by differentially expressed genes.

**FIGURE 7:**
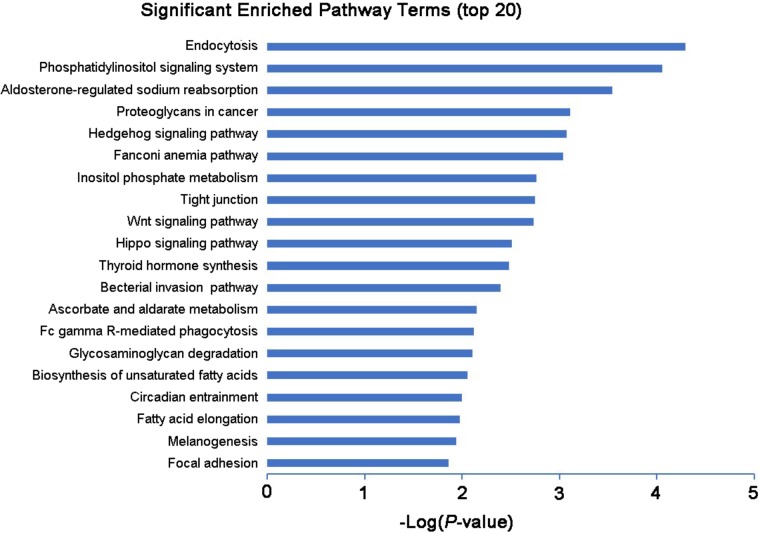
KEGG pathway analysis of the differentially expressed lncRNAs with top 20 enrichment scores.

Long non-coding RNAs have been identified to function as regulators by *cis-* and *trans-*patterns (Huang et al., 2016). To predict the target genes of differentially expressed lncRNAs, the co-expressed neighboring coding genes located within 10 kb of these lncRNAs were selected for analysis. A total of 27 coding genes corresponding to 29 lncRNAs were predicted (*cis*-regulation). Additionally, 114 lncRNAs were also identified to indirectly regulate the expression of 109 distant genes through binding miRNAs (*trans*-regulation). All these *cis*- and *trans*-targets were predicted and listed in **Supplementary Table S5**. The GO enrichment analyses indicated that these target genes were significantly associated with BP in membrane raft organization, membrane organization, membrane assembly and regulation of signal transduction, CC in intracellular organelle, dihydrolipoyl dehydrogenase complex, tricarboxylic acid cycle enzyme complex andintracellular part, MF in binding, cytoskeletal protein binding, and histone threonine kinase activity (**Figure 6** and **Supplementary Table S6**). The KEGG pathway enrichment analyses showed that these targets were also significantly enriched in hedgehog signaling pathway, tight junction, and Wnt signaling pathway (**Figure 7** and **Supplementary Table S7**).

## Discussion

Although some advances were achieved in biology, pathogenicity, and genetic characterization of *Cryptosporidium* (McDonald et al., 2001; Lee et al., 2017), no effective measures were developed to control cryptosporidiosis. The key challenge is that the interaction mechanism of host-*Cryptosporidium* has not been fully understood and appreciated. Unveiling the nature of host non-coding RNA world (e.g., lncRNA and miRNA) in last decades provided novel targets and strategies for preventing and treating infections of Theiler’s virus and *Salmonella* inflammatory bowel disease (IBD), diabetes, and multiple sclerosis in animals and humans (Atianand and Fitzgerald, 2014). In the present study, we systemically investigated the expression profiles of lncRNAs and mRNAs in HCT-8 cells infected with *C. parvum* IId subtype using microarray.

A total of 1,349 mRNAs were differentially expressed after the infection of *C. parvum* IId subtype. Among them, several inflammatory factors, e.g., IL-8, PTGS2, TCL-4, and CCL5 (RANTES), were up-regulated, and some genes associated with the cell proliferation and apoptosis were also significantly differentially expressed, e.g., up-regulated genes of thymidylate kinase, Cyclin A2, TM4SF1, IL1RN, Bcl2, and DUSP4, and down-regulated genes of Cyclin D1, Cyclin G2, BTG1, LAMB1, and LGALS1. These findings were consistent with previous studies using HCT-8 cells infected with *C. parvum* Iowa stain (IIaA15G2R1; Deng et al., 2004; Mele et al., 2004; Liu et al., 2009; Yang et al., 2015). Previous studies indicated that these genes would be involved in processes of inflammation, anti-apoptosis effect, and initiation and regulation of mucosal response during *C. parvum*, suggesting the important role of these genes within the interaction of host-*Cryptosporidium*. However, divergent expression patterns of mRNAs were also observed in HCT-8 cells infected with two subtypes of *C. parvum*. For example, the cell proliferation-related gene stratifin was down-regulated in our study, while it was up-regulated after the infection of *C. parvum* Iowa stain (Deng et al., 2004). Furthermore, the opposite trend of mRNA expression was detected for some negatively regulated genes (e.g., MT1B, MT1X, and MT1G) of apoptosis during infections of two different *C. parvum* subtypes (Liu et al., 2009), suggesting different pathogenic mechanisms of two subtypes.

Additionally, a total of 821 lncRNAs were found to be differentially expressed after the infection of *C. parvum* IIdA19G1. Co-expression and target prediction revealed 27 coding genes *cis*-regulated by 29 lncRNAs and 109 mRNAs *trans*-regulated by 114 lncRNAs. Function prediction of these differentially expressed transcripts was mainly involved in various pathways related to the infection and pathogenicity of *Cryptosporidium*, e.g., hedgehog signaling pathway, Wnt signaling pathway, and tight junction. In previous studies, the integrity of tight junction Zonula-Occludens-1 (ZO-1) was disrupted by *Cryptosporidium* infection (Buret et al., 2003). Wnt signaling plays a crucial role in the process of maintenance of intestinal epithelium. A particular hypothesis was undergoing investigated that Wnt signaling pathway was attenuated in intestinal epithelium infected with *C. parvum* (Zhang et al., 2016). The hedgehog signaling pathway showed negative effect within the Wnt signaling pathway and inhibition role in intestine proliferation (Katoh and Katoh, 2006). These findings suggested the possible regulating roles of host lncRNAs in these pathways during *Cryptosporidium* infection. Additionally, the qRT-PCR validation of 10 deregulated lncRNAs was consistent with the microarray data. Of them, the lncRNA XLOC_001265 was predicted to target RNF125. Previous studies have proved that mi-15b could regulate the Japanese *Encephalitis* Virus (JEV)-induced inflammatory cytokine (TNF-α, IL-1β, IL-6, CCL5, and IL-12p70) expression by targeting RNF125 (Zhu et al., 2015) in the JEV mouse model. Therefore, XLOC_001265 may be involved in the process of proinflammation caused by *C. parvum* IId subtype by regulating the expression of RNF125 because *C. parvum* had been proved to induce the expression of several inflammatory factors (IL-12, IL-17, IL-18, TNF-α, and TNF-γ; Ehigiator et al., 2005; Petry et al., 2010; O’Hara and Chen, 2011).

## Conclusion

The expression profiles of mRNA and lncRNA were investigated in the present study, and a total of 1,349 mRNAs and 821 lncRNAs were significantly differentially expressed in the HCT-8 cells infected with *C. parvum* IId subtype. Co-expression analysis revealed that these differentially expressed lncRNAs would potentially *cis*- and *trans-*regulate the expression of mRNAs during *C. parvum* infection. Findings in the present study would provide novel insights for exploring the control measures for diagnosis and control of cryptosporidiosis in humans and animals.

## Author Contributions

G-HZ conceived and designed the experiments. T-LL and X-CF performed the experiments and drafted the manuscript. T-LL, X-CF, Y-HL, Y-JY, and Y-LY analyzed the data. X-TW and L-XZ contributed to reagents and materials. All authors read and approved the final manuscript.

## Conflict of Interest Statement

The authors declare that the research was conducted in the absence of any commercial or financial relationships that could be construed as a potential conflict of interest.

## Footnotes

1 http://genespring-support.com/

2 http://kobas.cbi.pku.edu.cn/index.php

3 http://www.graphpad.com

4 http://www.cytoscape.org/

## Supplementary Material

FIGURE S1 Bioinformatics analysis of differentially expressed mRNAs and lncRNAs in HCT-8 cells infected with *Cryptosporidium parvum* IId subtype. **(A)** Hierarchical clustering plot showing expression profile of mRNAs. **(B)** Hierarchical clustering plot showing expression profile of lncRNAs. HCT-8 cells infected with *C. parvum* IIdA19G1 (O1–3) or without parasites (C1–3) were cultured for 24 h at RMPI 1640 medium and supplemented with 10% fetal bovine serum (FBS) under 5% CO2 at 37°C and collected for microarray analysis.

FIGURE S2 **(A)** The scatter plot showed the distributions of mRNAs. The values of *x* and *y* axes in the scatter plot were the normalized signal values of the samples (log2 scaled), and the *R* represents the correlation coefficient of the two group samples. The red point in the plot represents up-regulated mRNAs and lncRNAs, while the green point represents down-regulated mRNAs and lncRNAs. **(B)** The scatter plot showed the distributions of lncRNAs.

FIGURE S3 Comparison between microarray data and qRT-PCR results revealed a good correlation of two methods. The heights of the columns represent the fold changes computed from the microarray data and qRT-PCR results. The positive numbers represent up-regulated genes, while the negative numbers represent down-regulated genes.

TABLE S1 The primers of mRNAs and lncRNAs.

TABLE S2 The significantly differentially expressed mRNAs.

TABLE S3 The significantly differentially expressed lncRNAs.

TABLE S4 Co-expression of lncRNAs and mRNAs.

TABLE S5 The target genes predicted of lncRNAs.

TABLE S6 Go enrichment analysis of mRNAs and lncRNAs.

TABLE S7 KEGG pathway analysis of mRNAs and lncRNAs.

## References

[B1] Abrahamsen M. S. Schroeder A. A. Lancto C. A. (1996). Differential mRNA display analysis of gene expression in *Cryptosporidium parvum*-infected HCT-8 cells. *J. Eukaryot. Microbiol.* 43 80S–81S. 10.1111/j.1550-7408.1996.tb05008.x 8822873

[B2] Atianand M. K. Fitzgerald K. A. (2014). Long non-coding RNAs and control of gene expression in the immune system. *Trends Mol. Med.* 20 623–631. 10.1016/j.molmed.2014.09.002 25262537 PMC4252818

[B3] Broadbent K. M. Broadbent J. C. Ribacke U. Wirth D. Rinn J. L. Sabeti P. C. (2015). Strand-specific RNA sequencing in *Plasmodium falciparum* malaria identifies developmentally regulated long non-coding RNA and circular RNA. *BMC Genomics* 16:454. 10.1186/s12864-015-1603-4 26070627 PMC4465157

[B4] Buret A. G. Chin A. C. Scott K. G. (2003). Infection of human and bovine epithelial cells with *Cryptosporidium andersoni* induces apoptosis and disrupts tight junctional ZO-1: effects of epidermal growth factor. *Int. J. Parasitol.* 33 1363–1371. 10.1016/S0020-7519(03)00138-3 14527519

[B5] Cai M. Guo Y. Pan B. Li N. Wang X. Tang C. (2017). Longitudinal monitoring of Cryptosporidium species in pre-weaned dairy calves on five farms in Shanghai. China. *Vet. Parasitol.* 241 14–19. 10.1016/j.vetpar.2017.05.005 28579024

[B6] Checkley W. White A. C. Jr. Jaganath D. Arrowood M. J. Chalmers R. M. Chen X. M. (2015). A review of the global burden, novel diagnostics, therapeutics, and vaccine targets for *Cryptosporidium*. *Lancet Infect. Dis.* 15 85–94. 10.1016/S1473-3099(14)70772-8 25278220 PMC4401121

[B7] Cui Z. Wang R. Huang J. Wang H. Zhao J. Luo N. (2014). Cryptosporidiosis caused by *Cryptosporidium parvum* subtype IIdA15G1 at a dairy farm in Northwestern China. *Parasit. Vectors* 7:529. 10.1186/s13071-014-0529-z 25430474 PMC4254006

[B8] Delafosse A. Chartier C. Dupuy M. C. Dumoulin M. Pors I. Paraud C. (2015). *Cryptosporidium parvum* infection and associated risk factors in dairy calves in western France. *Prev. Vet. Med.* 118 406–412. 10.1016/j.prevetmed.2015.01.005 25623968 PMC7172863

[B9] Deng M. Lancto C. A. Abrahamsen M. S. (2004). *Cryptosporidium parvum* regulation of human epithelial cell gene expression. *Int. J. Parasitol.* 34 73–82. 10.1016/j.ijpara.2003.10.001 14711592

[B10] Deshpande A. P. Jones B. L. Connelly L. Pollock K. G. Brownlie S. Alexander C. L. (2015). Molecular characterization of *Cryptosporidium parvum* isolates from human cryptosporidiosis cases in Scotland. *Parasitology* 142 318–325. 10.1017/S0031182014001346 25244937

[B11] Ehigiator H. N. Romagnoli P. Borgelt K. Fernandez M. McNair N. Secor W. E. (2005). Mucosal cytokine and antigen-specific responses to *Cryptosporidium parvum* in IL-12p40 KO mice. *Parasite Immunol.* 27 17–28. 10.1111/j.1365-3024.2005.00736.x 15813719

[B12] Feng Y. Li N. Roellig D. M. Kelley A. Liu G. Amer S. (2017). Comparative genomic analysis of the IId subtype family of *Cryptosporidium parvum*. *Int. J. Parasitol.* 47 281–290. 10.1016/j.ijpara.2016.12.002 28192123 PMC5774651

[B13] Feng Y. Torres E. Li N. Wang L. Bowman D. Xiao L. (2013). Population genetic characterisation of dominant *Cryptosporidium parvum* subtype IIaA15G2R1. *Int. J. Parasitol.* 43 1141–1147. 10.1016/j.ijpara.2013.09.002 24126186

[B14] Feng Y. Xiao L. (2017). Molecular epidemiology of Cryptosporidiosis in China. *Front. Microbiol.* 8:1701 10.3389/fmicb.2017.01701 PMC5592218 28932217

[B15] Fletcher S. M. Stark D. Harkness J. Ellis J. (2012). Enteric protozoa in the developed world: a public health perspective. *Clin. Microbiol. Rev.* 25 420–449. 10.1128/CMR.05038-11 22763633 PMC3416492

[B16] Gookin J. L. Nordone S. K. Argenzio R. A. (2002). Host responses to *Cryptosporidium* infection. *J. Vet. Intern. Med.* 16 12–21. 10.1111/j.1939-1676.2002.tb01602.x 11822801

[B17] Guerrant D. I. Moore S. R. Lima A. A. Patrick P. D. Schorling J. B. Guerrant R. L. (1999). Association of early childhood diarrhea and cryptosporidiosis with impaired physical fitness and cognitive function four-seven years later in a poor urban community in northeast Brazil. *Am. J. Trop. Med. Hyg.* 61 707–713. 10.4269/ajtmh.1999.61.707 10586898

[B18] He Y. Meng X. M. Huang C. Wu B. M. Zhang L. Lv X. W. (2014). Long noncoding RNAs: novel insights into hepatocelluar carcinoma. *Cancer Lett.* 344 20–27. 10.1016/j.canlet.2013.10.021 24183851

[B19] Holland R. E. (1990). Some infectious causes of diarrhea in young farm animals. *Clin. Microbiol. Rev.* 3 345–375. 10.1128/CMR.3.4.345 2224836 PMC358168

[B20] Holubová N. Sak B. Horčičková M. Hlásková L. Květoňová D. Menchaca S. (2016). *Cryptosporidium avium* n. sp. (Apicomplexa: Cryptosporidiidae) in birds. *Parasitol. Res.* 115 2243–2251. 10.1007/s00436-016-4967-8 26905074 PMC4864505

[B21] Hu Y. Feng Y. Huang C. Xiao L. (2014). Occurrence, source, and human infection potential of *Cryptosporidium* and *Enterocytozoon bieneusi* in drinking source water in Shanghai, China, during a pig carcass disposal incident. *Environ. Sci. Technol.* 48 14219–14227. 10.1021/es504464t 25383482 PMC5788171

[B22] Huang J. Yue D. Qi M. Wang R. Zhao J. Li J. (2014). Prevalence and molecular characterization of *Cryptosporidium* spp. and *Giardia duodenalis* in dairy cattle in Ningxia, northwestern China. *BMC Vet. Res.* 10:292. 10.1186/s12917-014-0292-6 25488627 PMC4268852

[B23] Huang M. Zhong Z. Lv M. Shu J. Tian Q. Chen J. (2016). Comprehensive analysis of differentially expressed profiles of lncRNAs and circRNAs with associated co-expression and ceRNA networks in bladder carcinoma. *Oncotarget* 7 47186–47200. 10.18632/oncotarget.9706 27363013 PMC5216934

[B24] Insulander M. Silverlås C. Lebbad M. Karlsson L. Mattsson J. G. Svenungsson B. (2013). Molecular epidemiology and clinical manifestations of human cryptosporidiosis in Sweden. *Epidemiol. Infect.* 141 1009–1020. 10.1017/S0950268812001665 22877562 PMC9151846

[B25] Jezkova J. Horcickova M. Hlaskova L. Sak B. Kvetonova D. Novak J. (2016). *Cryptosporidium testudinis* sp. n., *Cryptosporidium ducismarci* Traversa, 2010 and *Cryptosporidium tortoise* genotype III (Apicomplexa: Cryptosporidiidae) in tortoises. *Folia Parasitol.* 63:2016.035. 10.14411/fp.2016.035 27827334

[B26] Josset L. Tchitchek N. Gralinski L. E. Ferris M. T. Eisfeld A. J. Green R. R. (2014). Annotation of long non-coding RNAs expressed in collaborative cross founder mice in response to respiratory virus infection reveals a new class of interferon-stimulated transcripts. *RNA Biol.* 11 875–890. 10.4161/rna.29442 24922324 PMC4179962

[B27] Katoh Y. Katoh M. (2006). Hedgehog signaling pathway and gastrointestinal stem cell signaling network (review). *Int. J. Mol. Med.* 18 1019–1023. 10.3892/ijmm.18.6.1019 17089004

[B28] Koinari M. Lymbery A. J. Ryan U. M. (2014). *Cryptosporidium* species in sheep and goats from Papua New Guinea. *Exp. Parasitol.* 141 134–137. 10.1016/j.exppara.2014.03.021 24703974

[B29] Kváč M. Havrdová N. Hlásková L. Daňková T. Kanděra J. Ježková J. (2016). *Cryptosporidium proliferans* n. sp. (apicomplexa: cryptosporidiidae): molecular and biological evidence of cryptic species within gastric *Cryptosporidium* of Mammals. *PLoS One* 11:e0147090. 10.1371/journal.pone.0147090 26771460 PMC4714919

[B30] Lee S. Harwood M. Girouard D. Meyers M. J. Campbell M. A. Beamer G. (2017). The therapeutic efficacy of azithromycin and nitazoxanide in the acute pig model of *Cryptosporidium hominis*. *PLoS One* 12:e0185906. 10.1371/journal.pone.0185906 28973041 PMC5626496

[B31] Lee S. Kopp F. Chang T. C. Sataluri A. Chen B. Sivakumar S. (2016). Noncoding RNA NORAD regulates genomic stability by sequestering PUMILIO proteins. *Cell* 164 69–80. 10.1016/j.cell.2015.12.017 26724866 PMC4715682

[B32] Li J. Lin X. Zhang L. Qi N. Liao S. Lv M. (2015). Molecular characterization of *Cryptosporidium* spp. in domestic pigeons (Columba livia domestica) in Guangdong Province, Southern China. *Parasitol. Res.* 114 2237–2341. 10.1007/s00436-015-4415-1 25773186

[B33] Liu J. Deng M. Lancto C. A. Abrahamsen M. S. Rutherford M. S. Enomoto S. (2009). Biphasic modulation of apoptotic pathways in *Cryptosporidium parvum*-infected human intestinal epithelial cells. *Infect. Immun.* 77 837–849. 10.1128/IAI.00955-08 19075026 PMC2632021

[B34] McDonald A. C. Mac-Kenzie W. R. Addiss D. G. Gradus M. S. Linke G. Zembrowski E. (2001). *Cryptosporidium parvum*-specific antibody responses among children residing in Milwaukee during the 1993 waterborne outbreak. *J. Infect. Dis.* 183 1373–1379. 10.1086/319862 11294669

[B35] Mele R. Gomez Morales M. A. Tosini F. Pozio E. (2004). *Cryptosporidium parvum* at different developmental stages modulates host cell apoptosis *in vitro*. *Infect. Immun.* 72 6061–6067. 10.1128/IAI.72.10.6061-6067.2004 15385510 PMC517591

[B36] Mi R. Wang X. Li C. Huang Y. Zhou P. Li Z. (2013). Prevalence and genetic characterization of *Cryptosporidium* in yaks in Qinghai Province of China. *PLoS One* 8:e74985. 10.1371/journal.pone.0074985 24086416 PMC3781125

[B37] Ming Z. Gong A. Y. Wang Y. Zhang X. T. Li M. Mathy N. W. (2017). Involvement of *Cryptosporidium parvum* Cdg7_FLc_1000 RNA in the attenuation of intestinal epithelial cell migration via trans-suppression of host cell SMPD3. *J. Infect. Dis.* 217 122–133. 10.1093/infdis/jix392 28961856 PMC5853865

[B38] Mondal D. Haque R. Sack R. B. Kirkpatrick B. D. Petri W. Jr. (2009). Attribution of malnutrition to cause-specific diarrheal illness: evidence from a prospective study of preschool children in Mirpur, Dhaka, Bangladesh. *Am. J. Trop. Med. Hyg.* 80 824–826. 19407131 PMC3410540

[B39] Nakamura A. A. Meireles M. V. (2015). *Cryptosporidium* infections in birds–a review. *Rev. Bras. Parasitol. Vet.* 24 253–267. 10.1590/S1984-29612015063 26444057

[B40] Nguyen S. T. Fukuda Y. Tada C. Sato R. Huynh V. V. Nguyen D. T. (2013). Molecular characterization of *Cryptosporidium* in pigs in central Vietnam. *Parasitol. Res.* 112 187–192. 10.1007/s00436-012-3124-2 23052759

[B41] O’Hara S. P. Chen X. M. (2011). The cell biology of *Cryptosporidium* infection. *Microbes Infect.* 13 721–730. 10.1016/j.micinf.2011.03.008 21458585 PMC3130844

[B42] Okazaki Y. Furuno M. Kasukawa T. Adachi J. Bono H. Kondo S. (2002). Analysis of the mouse transcriptome based on functional annotation of 60,770 full-length cDNAs. *Nature* 420 563–573. 10.1038/nature01266 12466851

[B43] Pawar H. Pai K. Patole M. S. (2017). A novel protein coding potential of long intergenic non-coding RNAs (lincRNAs) in the kinetoplastid protozoan parasite *Leishmania* major. *Acta Trop.* 167 21–25. 10.1016/j.actatropica.2016.12.012 27988178

[B44] Petry F. Jakobi V. Tessema T. S. (2010). Host immune response to *Cryptosporidium parvum* infection. *Exp. Parasitol.* 126 304–309. 10.1016/j.exppara.2010.05.022 20685209

[B45] Qi M. Z. Fang Y. Q. Wang X. T. Zhang L. X. Wang R. J. Du S. Z. (2015). Molecular characterization of *Cryptosporidium* spp. in pre-weaned calves in Shaanxi Province, north-western China. *J. Med. Microbiol.* 64 111–116. 10.1099/jmm.0.079327-0 25385243

[B46] Ryan U. Fayer R. Xiao L. (2014). *Cryptosporidium* species in humans and animals: current understanding and research needs. *Parasitology* 143 1667–1685. 10.1017/S0031182014001085 25111501

[B47] Ryan U. Paparini A. Tong K. Yang R. Gibson-Kueh S. O’Hara A. (2015). *Cryptosporidium huwi* n. sp. (Apicomplexa: eimeriidae) from the guppy (*Poecilia reticulata*). *Exp. Parasitol.* 150 31–35. 10.1016/j.exppara.2015.01.009 25637783

[B48] Squire S. A. Ryan U. (2017). *Cryptosporidium* and *Giardia* in Africa: current and future challenges. *Parasit. Vectors* 10:195. 10.1186/s13071-017-2111-y 28427454 PMC5397716

[B49] Wang R. Zhang L. Axén C. Bjorkman C. Jian F. Amer S. (2014). *Cryptosporidium parvum* IId family: clonal population and dispersal from Western Asia to other geographical regions. *Sci. Rep.* 4:4208. 10.1038/srep04208 24572610 PMC3936226

[B50] Wang Y. Gong A. Y. Ma S. Chen X. Strauss-Soukup J. K. Chen X. M. (2017b). Delivery of parasite Cdg7_Flc_0990 RNA transcript into intestinal epithelial cells during *Cryptosporidium parvum* infection suppresses host cell gene transcription through epigenetic mechanisms. *Cell Microbiol.* 19 636–643. 10.1111/cmi.12760 28655069 PMC5638686

[B51] Widmer G. Lee Y. Hunt P. Martinelli A. Tolkoff M. Bodi K. (2012). Comparative genome analysis of two *Cryptosporidium parvum* isolates with different host range. *Infect. Genet. Evol.* 12 1213–1221. 10.1016/j.meegid.2012.03.027 22522000 PMC3372781

[B52] Xiao L. (2010). Molecular epidemiology of cryptosporidiosis: an update. *Exp. Parasitol.* 124 80–89. 10.1016/j.exppara.2009.03.018 19358845

[B53] Yang Z. Fu Y. Gong P. Zheng J. Liu L. Yu Y. (2015). Bovine TLR2 and TLR4 mediate *Cryptosporidium parvum* recognition in bovine intestinal epithelial cells. *Microb. Pathog.* 85 29–34. 10.1016/j.micpath.2015.05.009 26048276

[B54] Zahedi A. Durmic Z. Gofton A. W. Kueh S. Austen J. Lawson M. (2017). *Cryptosporidium homai* n. sp. (Apicomplexa: Cryptosporidiiae) from the guinea pig (*Cavia porcellus*). *Vet. Parasitol.* 245 92–101. 10.1016/j.vetpar.2017.08.014 28969844

[B55] Zhang H. Zhu C. Zhao Y. Li M. Wu L. Yang X. (2015). Long non-coding RNA expression profiles of hepatitis C virus-related dysplasia and hepatocellular carcinoma. *Oncotarget* 6 43770–43778. 10.18632/oncotarget.6087 26540467 PMC4791265

[B56] Zhang X. X. Tan Q. D. Zhou D. H. Ni X. T. Liu G. X. Yang Y. C. (2015). Prevalence and molecular characterization of *Cryptosporidium* spp. in dairy cattle, northwest China. *Parasitol. Res.* 114 2781–2787. 10.1007/s00436-015-4537-5 26002827

[B57] Zhang X. T. Gong A. Y. Wang Y. Chen X. Lim S. S. Dolata C. E. (2016). *Cryptosporidium parvum* infection attenuates the ex vivo propagation of murine intestinal enteroids. *Physiol. Rep.* 4:e13060. 10.14814/phy2.13060 28039407 PMC5210379

[B58] Zhao G. H. Gong A. Y. Wang Y. Zhang X. T. Li M. Mathy N. W. (2018). Nuclear delivery of parasite Cdg2_FLc_0220 RNA transcript to epithelial cells during *Cryptosporidium parvum* infection modulates host gene transcription. *Vet. Parasitol.* 251 27–33. 10.1016/j.vetpar.2017.12.015 29426472 PMC5808497

[B59] Zhao Z. Wang R. Zhao W. Qi M. Zhao J. Zhang L. (2015). Genotyping and subtyping of *Giardia* and *Cryptosporidium* isolates from commensal rodents in China. *Parasitology* 142 800–806. 10.1017/S0031182014001929 25579244

[B60] Zhou R. Feng Y. Chen X. M. (2014). Non-coding RNAs in epithelial immunity to *Cryptosporidium* infection. *Parasitology* 141 1233–1243. 10.1017/S0031182014000614 24828969 PMC4327859

[B61] Zhou R. Hu G. Liu J. Gong A. Y. Drescher K. M. Chen X. M. (2009). NF-kappaB p65-dependent transactivation of miRNA genes following *Cryptosporidium parvum* infection stimulates epithelial cell immune responses. *PLoS Pathog.* 5:e1000681. 10.1371/journal.ppat.1000681 19997496 PMC2778997

[B62] Zhu B. Ye J. Nie Y. Ashraf U. Zohaib A. Duan X. (2015). MicroRNA-15b Modulates Japanese *Encephalitis* Virus-Mediated inflammation via targeting RNF125. *J. Immunol.* 195 2251–2262. 10.4049/jimmunol.1500370 26202983

