# Supplementary figures and images for: Expression Profiles of mRNA and lncRNA in HCT-8 Cells Infected With Cryptosporidium parvum IId Subtype
Source: Front Microbiol. 2018 Jun 27;9:1409. doi: 10.3389/fmicb.2018.01409 (PMC6036261; doi:10.3389/fmicb.2018.01409)

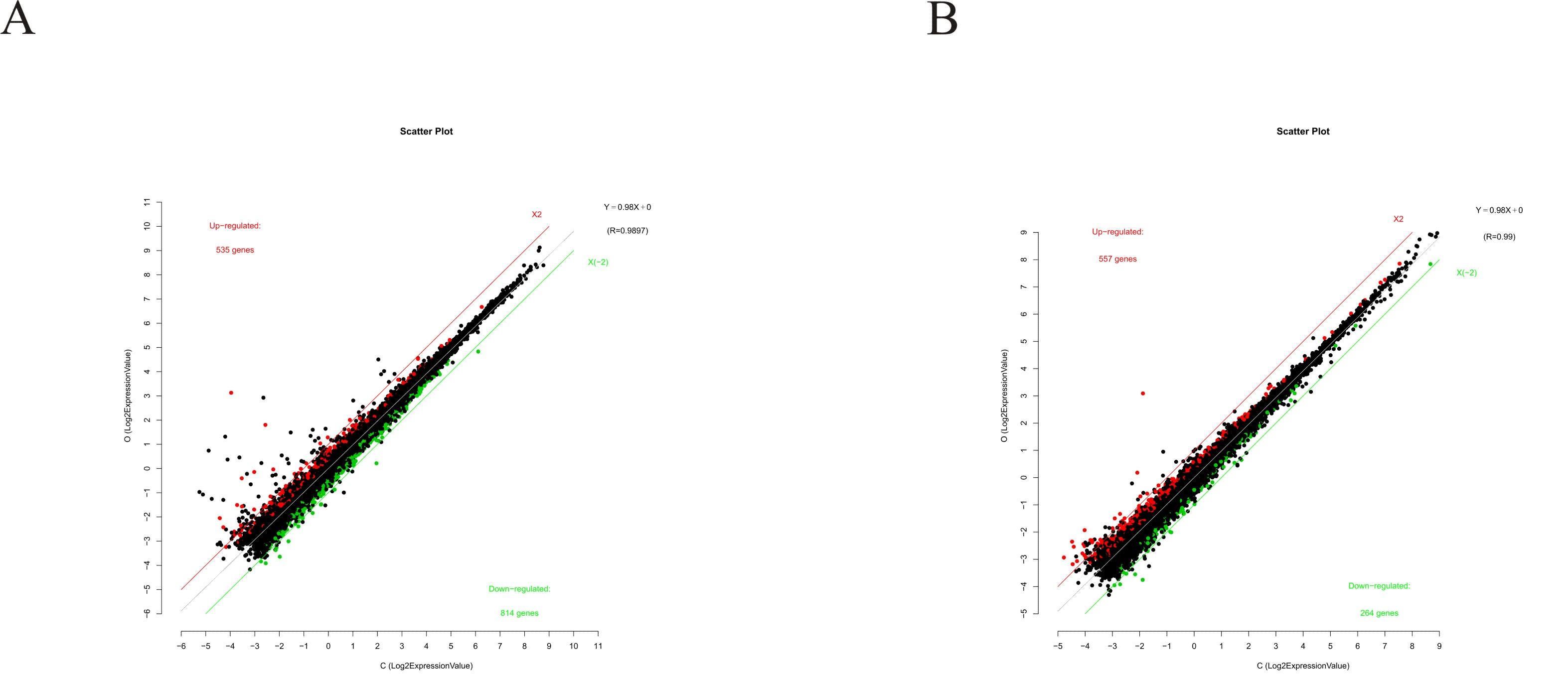

Supplement: FIGURE S2 — (A) The scatter plot showed the distributions of mRNAs. The values of x and y axes in the scatter plot were the normalized signal values of the samples (log2 scaled), and the R represents the correlation coefficient of the two group samples. The red point in the plot represents up-regulated mRNAs and lncRNAs, while the green point represents down-regulated mRNAs and lncRNAs. (B) The scatter plot showed the distributions of lncRNAs. [file Image_2.TIF]

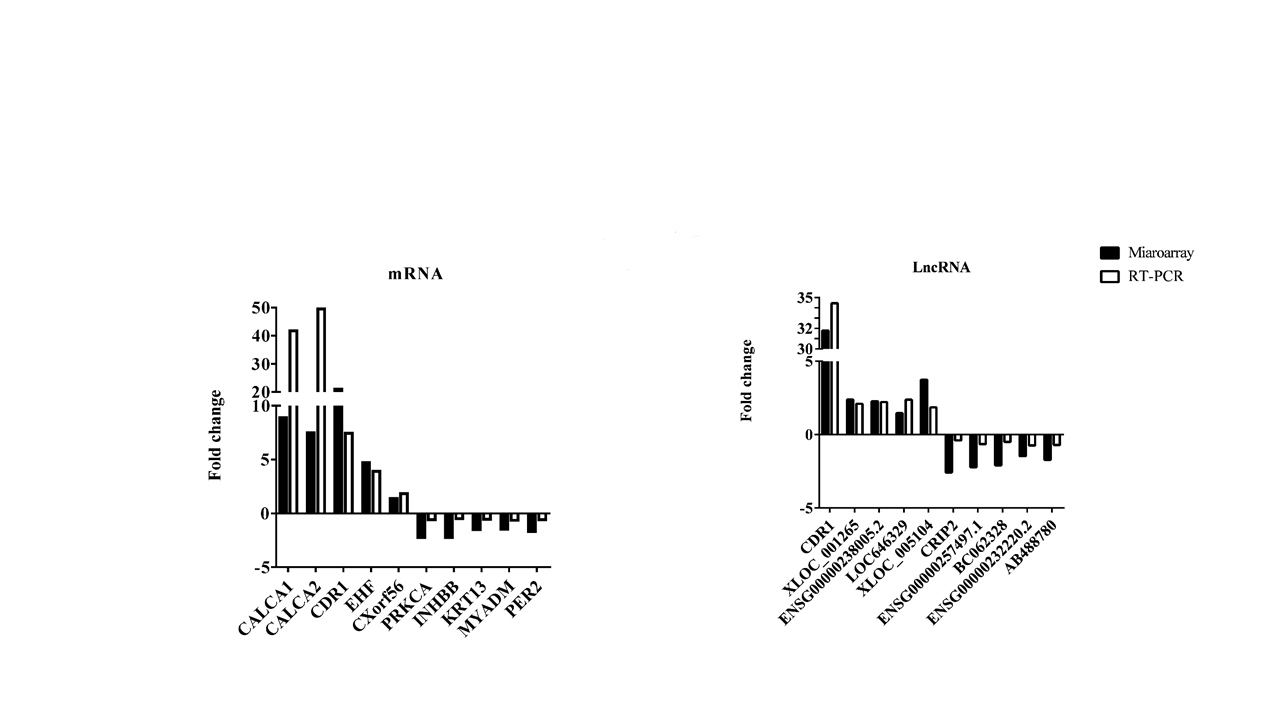

Supplement: FIGURE S3 — Comparison between microarray data and qRT-PCR results revealed a good correlation of two methods. The heights of the columns represent the fold changes computed from the microarray data and qRT-PCR results. The positive numbers represent up-regulated genes, while the negative numbers represent down-regulated genes. [file Image_3.TIF]
